# Quantification of Low Amounts of Zoledronic Acid by HPLC-ESI-MS Analysis: Method Development and Validation

**DOI:** 10.3390/ijms23115944

**Published:** 2022-05-25

**Authors:** Anca-Roxana Petrovici, Mihaela Silion, Natalia Simionescu, Rami Kallala, Mariana Pinteala, Stelian S. Maier

**Affiliations:** 1 Centre of Advanced Research in Bionanoconjugates and Biopolymers, “Petru Poni” Institute of Macromolecular Chemistry, 41A Grigore Ghica Voda Alley, 700487 Iasi, Romania; petrovici.anca@icmpp.ro (A.-R.P.); pinteala@icmpp.ro (M.P.); 2 Physics of Polymers and Polymeric Materials Department, “Petru Poni” Institute of Macromolecular Chemistry, 41A Grigore Ghica Voda Alley, 700487 Iasi, Romania; silion.mihaela@icmpp.ro; 3 Corthotec Limited, 130 Wood Street, London EC2V 6DL, UK; admin@corthotec.com; 4 Polymers Research Center, “Gheorghe Asachi” Technical University of Iasi, 73 Dimitrie Mangeron Blvd., 700050 Iasi, Romania

**Keywords:** zoledronic acid (ZA), calcium sulfate hemihydrate, solid inorganic matrix, HPLC-ESI-MS analysis, extracted ion chromatogram (EIC), ZA-calcium complexes, method development and validation

## Abstract

Zoledronic acid (ZA) is used in the treatment of various bone pathologies, but it forms complexes with calcium ions present in body fluids, decreasing ZA bioavailability. Thereby, the study first describes the identification of ZA-calcium complexes that form in calcium-rich environments, in order to establish the bioavailable ZA concentration. Then, a new method for quantification of low ZA amounts in milieus that mimics in vivo conditions by using simulated body fluid and calcium sulfate hemihydrate was described. Almost all analytical methods of ZA quantification described in the literature require compound derivatization. At very low concentrations, derivatization is prone to analyte loss, therefore compromising the analytical results. In our study, we avoided ZA derivatization by using a high-performance liquid chromatography and electrospray ionization mass spectrometry (HPLC-ESI-MS) system, conducting the investigation based on the fragmentation mass extracted ion chromatograms specific to the ZA protonated form. The method was validated by selectivity, precision, accuracy, linearity, signal to noise ratio, and limit of detection and limit of quantification calculation. Experimentally, this method can detect ranges of 0.1–0.5 ng/mL and precisely quantify ZA concentrations as low as 0.1 ng/mL. This method could provide the basis for quantifying low amounts of ZA in the blood during long-term administration.

## 1. Introduction

Zoledronic acid (IUPAC name (1-Hydroxy-2-imidazol-1-yl-phosphonoethyl) phosphonic acid, (ZA)) is known as a third-generation bisphosphonate [1]. Bisphosphonates represent an important family of drugs used in the treatment of various bone and calcium-related pathologies, such as cancer [2], hypercalcemia, tumor-induced hypercalcemia [1], Paget’s disease [3] and osteoporosis [4,5]. Bisphosphonates exhibit powerful inhibitory effects on osteoclastic bone resorption [6], due to their affinity to bone minerals. In physico-chemical terms, they firmly bind to calcium phosphate, inhibiting the processes of crystals’ growth, aggregation and dissolution [4]. At low doses, bisphosphonates do not adversely affect bone formation and mineralization and do not have a notable impact on renal function, providing an improved ratio of antiresorptive effects versus renal adverse effects [7].

However, long-term administration and high ZA doses often lead to osteonecrosis of the jaw [8,9]. Di Vito et al., in 2020 [10], evaluated the biological effects of different ZA concentrations on the in vitro model of periodontal ligament stem cells. The results showed that cell viability remained high when ZA concentrations between 27.2 and 272.09 ng/mL were used [10]. Furthermore, concentrations higher than 408.13 ng/mL impaired cells’ viability by inducing apoptosis [10,11], highlighting the opposite effects of ZA treatment depending on the dose administered. However, there is evidence that medication-related osteonecrosis of the jaw could be alleviated by different therapeutical approaches, including the administration of exosomes derived from mesenchymal stem cells, as reviewed in [12].

In contrast to the side effects generated by ZA treatment, there are also beneficial effects reported by some research groups. For example, Munoz et al. [13] reported in a study published in 2021 that ZA not only acts on the bones, but also binds to other systems in the body. By fluorescently labeling a bisphosphonate compound, they established that the compound is internalized in vivo by alveolar macrophages and large peritoneal macrophages [13]. Furthermore, they showed that one dose administration (in concentration of 272.09, 1360.45 or 2720.9 ng/mL ZA) boosted the immune response of immuno-suppressed patients in bacterial lung infections, which cause pneumonia [13].

The pharmacological function of bisphosphonates is determined by the P-C-P configuration, where two phosphate groups are covalently linked to a carbon atom [14]. Their exact mechanism of action was proved only recently [3]. The groups linked to the carbon atom of the P-C-P chain influence the pharmacokinetics, the mode of action and the strength of the drugs [3]. Chemically, they are all related to pyrophosphate, which is formed as a by-product of cellular metabolism during nucleotide synthesis. Pyrophosphate is a natural inhibitor of the bone mineralization process, which is balanced by alkaline phosphatase [3]. The bisphosphonates are formed by substituting one atom of oxygen with one carbon, and they are able to penetrate the bone solid mass and strongly attach to the bone minerals [3].

Due to the peculiar chemical nature of ZA, its chromatographic separation has proven challenging. Bisphosphonates do not have the strong chromophores typically used for UV detection in ordinary high-performance liquid chromatography (HPLC) methods. Various strategies for the determination of ZA in biological matrices were investigated, including optimization of sample preparation and derivatization procedures, separation using liquid or gas chromatography, and detection by mass spectrometry [7,15,16,17]. None of these approaches provided sufficient analytical reproducibility and sensitivity [7].

Veldboer et al. [15] developed a method for ZA quantification in urine and blood samples by derivatization with trimethylsilyl diazomethane, in order to produce ZA-tetramethyl phosphonate, which is less polar than its parent compound [18,19], and by using a stable isotope-labeled internal standard as calibration. For the chromatographic separation, they used a binary gradient generated by mixing (i) 95% ammonium acetate and 5% MeOH (10 mM, pH 7), and (ii) 95% MeOH and 5% ammonium acetate (10 mM, pH 7), on a ProntoSIL C18 UHC 330 column (3 μm, 30 mm × 3.0 mm) [15]. Legay et al. [7] developed a method for ZA quantification using an HPLC-reverse isotope dilution method, determining ZA concentrations from ^14^C total radioactivity by using a combination of antibody and tracer in a competitive radioimmunoassay. Later, Raina et al. [16] identified and quantified ZA by coupling ^14^C-ZA with a scintillation cocktail and using a scintillation counter. All these methods are time consuming due to additional sample processing [20] and may fail at very low concentrations of ZA.

Rao et al. [17] validated a method to determine ZA in a range of concentrations of 200 to 1000 μg/mL using an isocratic ion-pair RP-LC method. The separation was performed on a Waters XTerra RP 18, 250 mm × 4.6 mm, 5 μm column, using a mobile phase made by a solution of methanol:water solution (containing a mixture of 8 mM dipotassium hydrogen orthophosphate, 2 mM di-sodium hydrogen orthophosphate and 7 mM tetra-n-butyl ammonium hydrogen sulfate) (95:5, *v*/*v*), at a flow rate of 0.7 mL/min, and separation monitoring at 215 nm [17].

The results obtained and published in recent literature regarding ZA identification and quantification were satisfactory, but the methods used were not applicable and cannot be adapted to very low concentrations of ZA. Moreover, the concentrations investigated in the literature were very high [21,22,23,24], while in biological systems, the bioavailable ZA concentration, which is not cytotoxic, is lower than 272.09 ng/mL [10]. Therefore, in order to quantify ZA from biological matrices at non-toxic concentrations [25], it is necessary to develop new non-invasive methods specifically tailored for the active compound or organism investigated.

The aim of the present study was to develop and validate a high-performance liquid chromatography and electrospray ionization mass spectrometry (HPLC-ESI-MS) method for the characterization and quantification of ZA at low concentrations, released from a solid inorganic matrix, using an external calibration method and extracted ion chromatogram (EIC) analysis. ZA interacts with free calcium ions, forming insoluble complexes [26] and decreasing its bioavailability. By using the HPLC-ESI-MS technique we can identify ZA-calcium complexes, thus being able to determine and quantify very low amounts of ZA. Therefore, the developed HPLC–ESI–MS technique provides a powerful scientific basis for future in vitro and in vivo pharmacological studies.

## 2. Materials and Methods

### 2.1. Reagents

Zoledronic acid was purchased from Actavis Group PTC ehf. (Hafnarfirdi, Iceland) in sterile vials of 4 mg/5 mL concentration. Calcium sulfate hemihydrate, methanol HPLC grade and formic acid 98–100% were purchased from Merck Group (Darmstadt, Germany).

### 2.2. ZA-Ca^2+^ Complex Formation

In order to analyze the ZA-Ca^2+^ complex formation by ESI-MS, solutions of ZA:Ca at molar ratios of 2:1 and 1:1 were prepared. The calcium ions solutions were prepared in Milli-Q purified water and the mixed solutions were subjected to HPLC-ESI-MS analysis.

### 2.3. ZA Quantification Method—Development and Validation

#### 2.3.1. HPLC-ESI-MS Method Development

The analysis of ZA was performed using an Agilent 1200 Series HPLC system with diode array detector (DAD) coupled to an Agilent 6520 accurate-mass quadrupole time-of-flight (Q-TOF) mass spectrometer equipped with electrospray (ESI) source (Agilent Technologies, Inc., Santa Clara, CA, USA). The separation was carried out on a 150 × 4.5 mm, 5 µm Brownlee Analytical Amino column, with a pre-column (PerkinElmer, Inc., Waltham, MA, USA) at 25 °C. The mobile phase consisted of (i) 75% water with 10 mM formic acid, and (ii) 25% methanol with 10 mM formic acid, and isocratic elution was applied [19]. The flow rate was 0.8 mL/min, and 10 µL of samples was injected. The DAD acquisition wavelength was set in the range of 200–800 nm, and the separation process was monitored at 215 nm.

After DAD detector, the elute was split and 0.1 mL/min was directed to ESI/Q-TOF MS. The instrument was operated at an ionization voltage of +4000 V and source temperature of 325 °C. Nitrogen was used as nebulizer gas at 25 psi and as drying gas at a flow rate of 7 L/min. The full scan of ions ranging from *m*/*z* 50 to 2000 in the positive ion mode was used. The mass scale was calibrated using the standard calibration procedure and compounds provided by the manufacturer. Data were collected and processed using MassHunter Workstation Software Data Acquisition for 6200/6500 Series, version B.01.03 (Agilent Technologies, Inc., Santa Clara, CA, USA).

The HPLC and EIC chromatograms were obtained using the method described above. Retention time (Rt) of ZA was determined by injecting the standard curve concentrations. ZA concentrations were monitored using the extracted EIC peak characteristic of *m*/*z* 273 [M + H]^+^.

#### 2.3.2. Preparation of ZA Standard Solutions

The ZA standard curve solutions were prepared by using calcium sulfate hemihydrate solution in simulated body fluid (SBF). The SBF was prepared after a protocol described by Bayraktar and Tas [27]. In order to prepare the standard solutions, 2.12 mM calcium sulfate hemihydrate was suspended in 10 mL SBF and different ZA amounts were added. ZA concentrations of 0.1; 0.2; 0.3; 0.4 and 0.5 ng ZA/mL (0.37 × 10^−6^; 0.74 × 10^−6^; 1.1 × 10^−6^; 1.47 × 10^−6^ and 1.84 × 10^−6^ mM, respectively) were vigorously vortexed for 30 s and kept at 4 °C for 72 h. After three days, all the samples were filtered through 0.2 μm filters and subjected to HPLC-ESI-MS analysis using the conditions described in Section 2.3.1. The standard curve was plotted as EIC peak area (Y) versus ZA standard concentration (X).

#### 2.3.3. HPLC-ESI-MS Method Validation

The method used for ZA determination was validated using the following parameters: selectivity, precision, accuracy, linearity, signal to noise ratio, limit of detection and limit of quantification [28]. The method validation was performed using the ZA standard solutions described in Section 2.3.2.

##### Selectivity

The method used for separation is considered selective if the peaks corresponding to ZA do not interfere with other peaks in chromatograms, and if the peaks have the same morphology as in the standard samples prepared in the same conditions [28]. In order to determine the selectivity of the method, the calculations were performed using the chromatograms of the reference solution (a stock solution of ZA with a concentration of 0.1 ng/mL), of a sample solution (prepared using calcium sulfate hemihydrate in excess) and of a blank solution (containing no active compound). The selectivity factor (α) was calculated using the following formula:

$$\alpha =\frac{{\mathrm{Rt}}_{2}-{\;t}_{0}}{{\mathrm{Rt}}_{1}-{\;t}_{0}}$$

where Rt_1_ is the retention time for ZA in the reference solution; Rt_2_ is the retention time for ZA in the sample solution; and t_0_ is the retention time of non-retained compound from chromatogram.

##### Precision

The precision of a method represents the degree of agreement between the results of individual tests when the procedure is repeated for multiple sampling [28]. In order to determine the precision of the method, solutions with concentrations of 0.1; 0.2; 0.3; 0.4 and 0.5 ng ZA/mL were prepared, each solution was injected 6 times and the retention time was determined for each peak. SD and RSD% were calculated using the peak areas determined for each of the standards, as suggested by Rao [28].

##### Accuracy

The accuracy of the method was calculated considering the following protocol. A stock solution of 0.5 ng ZA/mL was chosen and proportions of 80, 100 and 120% of the concentration were prepared individually from standards, in the conditions described in Section 2.3.2. All three solutions were injected 6 times each into the HPLC system using the method described in Section 2.3.1. From the obtained chromatograms, the peak areas were determined and the concentration was calculated using the calibration curve, as suggested by Rao [28].

##### Linearity

The linearity of the method was calculated using the peak areas determined for the representative concentrations of standard ZA solutions (0.1; 0.2; 0.3; 0.4 and 0.5 ng ZA/mL), injected 6 times each on HPLC-MS system. The linearity of the response function was determined by representing the peak areas as function of concentration, using the standard curve equation with the corresponding correlation coefficient (R^2^). The concentrations of the standard ZA solutions were calculated using the equation of the standard curve. The calculated concentrations were represented as a function of the theoretical concentrations, therefore obtaining the linearity of the results. A calibration curve is considered to be linear if R^2^ is over 0.98 [28].

##### Signal-to-Noise Ratio

The signal-to-noise ratio (S/N) was calculated using the MassHunter Qualitative Analysis software (Agilent Technologies, Inc., Santa Clara, CA, USA) for ZA corresponding peak. By following the software’s instructions, we set the value of baseline noise 30 s before and after the analyzed ZA peak, and the S/N ratio was automatically calculated.

##### Limit of Detection and Limit of Quantification

Limit of detection (LOD) and limit of quantification (LOQ) were calculated based on the method described by Rao [28], using the following formulas:

LOD=3× S/N × Lowest concentration of the linearity sample

LOQ=10× S/N × Lowest concentration of the linearity sample

## 3. Results and Discussion

Treatment with low doses of ZA leads to beneficial effects on the body such as reducing lung infections or regenerating osteonecrosis of the jaw [10,13]. However, in order to evaluate low concentrations of ZA in systems such as cell cultures or clinical trials, it is necessary to develop chromatographic methods capable of quantifying such low concentrations. At the same time, it should be noted that ZA forms complexes with calcium ions present in the environment [26], so its bioavailability decreases, therefore decreasing its concentration compared to the amount administered.

Considering the fact that the clinical treatment with ZA is performed by intravenous administration, where calcium ions form insoluble calcium salts with the highly negatively charged ZA moiety [7,26], in our study the ZA monitoring and quantification by HPLC–ESI–MS technique were performed in a medium containing various amounts of Ca^2+^. In bone, the active form of ZA binds naturally to hydroxyapatite, providing a constant concentration of calcium and phosphate ions released in the system [29] and at the same time inhibiting osteoclast-mediated bone desorption [10]. Therefore, the quantification of the free ZA amount in a system must be performed after taking into account the calcium complexes’ formation in order to avoid interferences and to determine the exact amount of ZA.

### 3.1. ZA-Ca^2+^ Complex Formation

**Figure 1:**
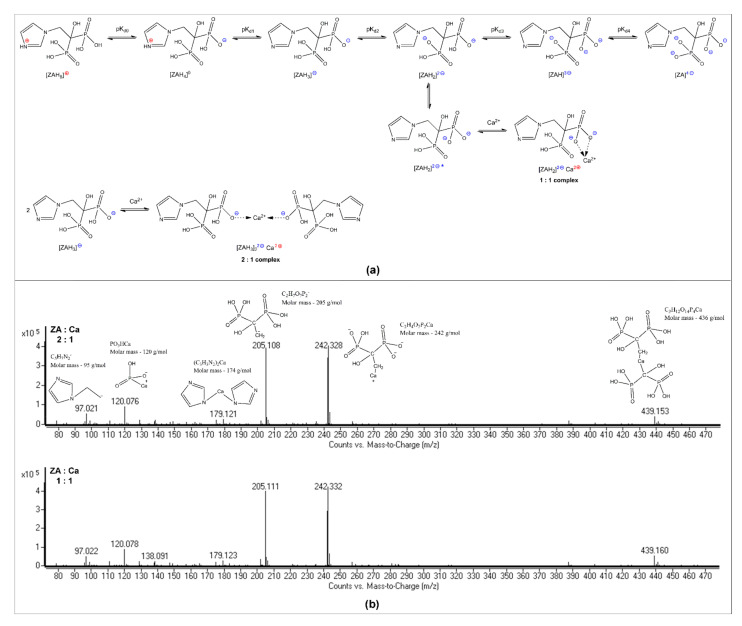
Complexes’ formation between Ca^2+^ and ZA: (**a**) ZA dissociation and Ca^2+^ complexation equilibria; (**b**) Confirmation of complexes’ formation by ESI-MS (ZA:Ca^2+^ molar ratios of 2:1 and 1:1).

In the ZA monitoring and quantification process, we have encountered various challenging situations when we tried to determine the exact amount of free ZA, so we concluded that we need to identify and quantify both the complexed chemical species and the free ZA amounts, respectively. Mostefa Side Larbi et al. [26] identified the existence of three complexes involved in the interaction between Ca^2+^ and ZA (ZA–Ca–ZA, ZA–Ca, Ca–ZA–Ca). The formation of the complexes is possible due to ionic interactions between Ca^2+^ and the negatively charged forms of dissociated ZA. By binding calcium, a strong rearrangement of the surrounding water molecules occurs, along with proton release or uptake. Furthermore, the affinity of calcium for each ZA site (Figure 1a) and therefore the formation of ZA–Ca–ZA, ZA–Ca or Ca–ZA–Ca complexes, is pH dependent.

In order to obtain ZA-Ca^2+^ complexes, we prepared solutions with ZA:Ca^2+^ molar ratios of 2:1 and 1:1. The ESI-MS spectrum revealed the formation of six principal species, which confirmed the complexes’ formation in both molar ratios used, of 2:1 and 1:1, respectively (Figure 1b).

**Figure 2:**
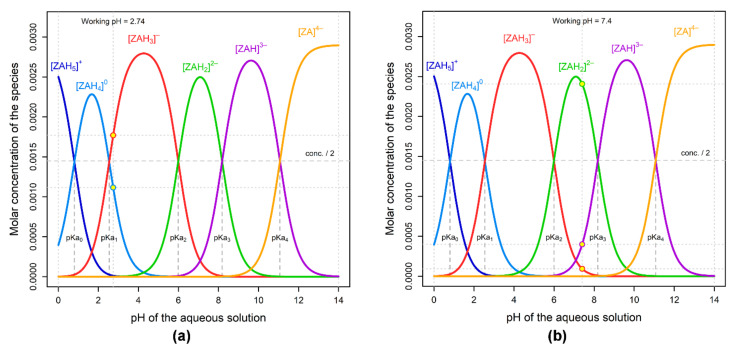
ZA speciation as a function of pH in the commercial aqueous solution of 0.0029 M (4 mg/5 mL, Actavis Group): (**a**) Working pH = 2.74; (**b**) Working pH = 7.4.

Figure 2 depicts the speciation of the ZA ionized forms as a pH function of the aqueous solutions. In order to perform the experiment, a concentration of 0.0029 M for ZA was considered, as calculated for the concentration of the commercial product, which is 4 mg ZA/ 5 mL. The considered values of pKa of zoledronic acid [30] were pKa0 = 0.8; pKa1 = 2.54; pKa2 = 5.99; pKa3 = 8.18; pKa4 = 11.07. Yellow points in Figure 2 indicate the abundance of the ZA ionic species: (a) in an aqueous solution having the commercial product’s pH (2.74); and (b) in the buffered solution of calcium sulfate (pH 7.4), at 25 °C. The commercial solution has a pH value near the second pKa value of ZA (2.74 vs. 2.54) and, therefore, it contains both mono- and bi-anionic forms of ZA in similar concentrations, which determine the predominant formation of 1:1 complexes (Figure 1a). An additional confirmation can be seen from fragments detected in MS spectra (Figure 1b) where we can observe that ZA-Ca-ZA and ZA-Ca complexes are formed and that the ZA-Ca complex is formed in a pH range between 7 and 9.5 [26], as the principal molecular species (70%). Due to the fact that we worked at pH 7, we were unable to observe the formation of the Ca-ZA-Ca complex, which forms only at pH values above 9.5 [26]. Working at neutral pH was chosen for two reasons. Firstly, the pH of the bone environment is close to neutral. Secondly, working at neutral pH avoided the irreversible damage of the chromatographic column by clogging, which occurs at alkaline values.

### 3.2. HPLC-ESI-MS Method Development

**Figure 3:**
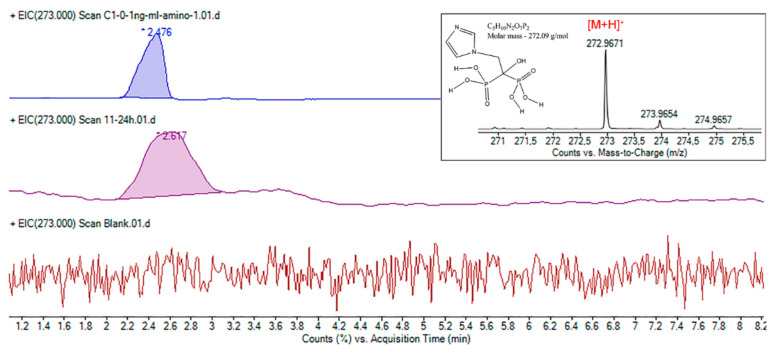
Representative extracted ion chromatograms (EIC) of active compound at *m*/*z* 273. Detail: ZA structure confirmation on amino column by HPLC-ESI-MS.

A major challenge in the process of ZA monitoring and quantification was choosing the column for separation. Due to the presence of two phospho-acid groups in the ZA structure, its polarity is too strong to retain on non-polar stationary phases such as the C8 or C18 column [1]. On the other hand, ZA’s ability to complex Ca^2+^ and the tendency to form multiple chemical species leads to undefined peaks with low intensities [15]. In this case, the amino columns can be considered and used in an anion-exchange mode separation. Being an ionic and highly polar molecule, ZA is retained on the amino columns that show a strong polar behavior induced by the negative dipole moment in the direction of the N atom of amino groups [31]. By injecting a standard solution prepared as in Section 2.3.2, we confirmed the ZA structure, as shown in Figure 3, by searching the *m*/*z* value of ZA in positive mode. The Rt for standard ZA was recorded at 2.315 min and the Rt for the ZA from samples was recorded at 2.617 min.

#### ZA Calibration Curve

**Figure 4:**
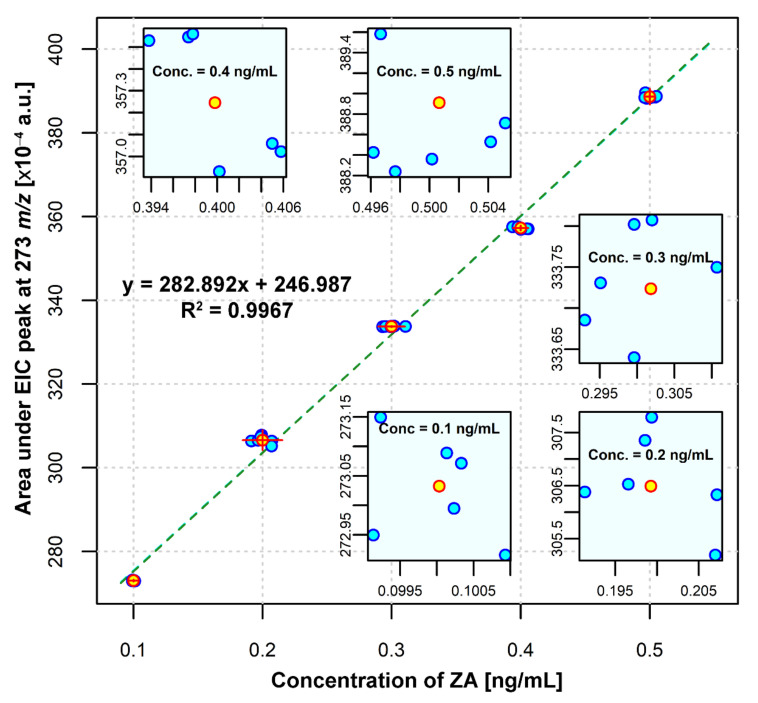
ZA calibration curve with Deming regression.

The calibration curve was established in the conditions described in Section 2.3.2, by using calcium sulfate hemihydrate in excess. After injecting the standard ZA solutions, six times each, into the HPLC-ESI-MS system, the HPLC chromatograms and EICs were obtained. ZA concentrations were monitored using the EIC peak area of *m*/*z* 273.00 corresponding to the protonated ZA [M + H]^+^ and the calibration curve was plotted as EIC peak area versus ZA standard concentration (Figure 4).

### 3.3. HPLC-ESI-MS Method Validation

#### 3.3.1. Selectivity

Usually, the selectivity factor is used to characterize how well chromatographic peaks are separated when peaks of more than one compound appear in chromatograms. In our case, we used the selectivity factor to identify how well the standards separate compared with samples. In order to determine the selectivity of the method, the chromatograms of a standard solution of 0.1 ng ZA/mL, a sample solution obtained using calcium sulfate hemihydrate in excess and a blank solution containing no active compound were used for calculation. The method used for separation is considered selective if the peaks corresponding to ZA do not interfere with other peaks present in chromatograms, and if the peaks have the same morphology as in the standard solutions prepared in the same conditions [28]. By applying the formula presented in Section 2.3.3—Selectivity, we obtained an α value of 1.13, which confers a good selectivity factor of the method.

#### 3.3.2. Precision

The precision of a method refers to the degree of agreement between individual test results when the procedure is repeated for multiple sampling [28]. The method’s precision was determined using the peak areas of six individual injections for each standard concentration, calculating the standard deviation (SD) and the relative standard deviation (RSD%) for each concentration. Table 1 presents the data obtained for the lowest ZA concentration of the standard curve (0.1 ng ZA/mL). SD and RSD% in the range of ±2% are characteristics of a precise method [28]. Values in Table 1 are lower than 0.09% and 0.03%, respectively, confirming the precision of our method.

#### 3.3.3. Accuracy

In order to determine the accuracy of the method, we considered the value of 0.5 ng ZA/mL as 100% concentration and we prepared proportions of 80% (0.4 ng ZA/mL), 100% (0.5 ng ZA/mL) and 120% (0.6 ng ZA/mL), as suggested by Rao [28] and described in Section 2.3.3—Accuracy. Six individual injections were performed for each solution, following the protocol described in Section 2.3.1, and the peak area for each sample was obtained by extraction of EIC of *m*/*z* 273 [M + H]^+^. The standard curve equation (Figure 4) was used to calculate the concentration, and all data are presented in Table 2. The calculated concentration for the 80% ZA sample was 0.39 ng ZA/mL; for the 100% ZA sample, it was 0.501 ng ZA/mL; and for the 120% ZA sample, it was 0.602 ng ZA/mL, confirming the accuracy of the method used for ZA quantification. In addition, SD and RSD% values were very good, confirming the precision of the method for all the determinations.

#### 3.3.4. Linearity

**Figure 5:**
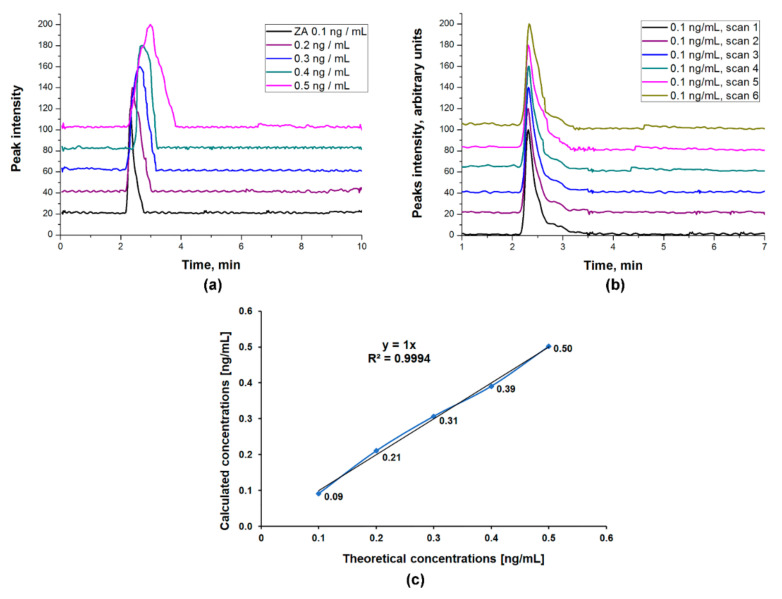
Linearity of the HPLC-ESI-MS method: (**a**) Chromatograms recorded for standard curve concentrations; (**b**) Chromatograms recorded for the 6 injection scans of 0.1 ng ZA/mL; (**c**) Linearity plot for ZA standards.

The linearity of the method was confirmed by the chromatograms recorded for all the concentrations of the ZA standard curve (0.1; 0.2; 0.3; 0.4 and 0.5 ng ZA/mL), injected six times each (Figure 5a,b). In order to calculate the linearity of the method, we plotted the calculated concentrations as a function of theoretical concentrations (Figure 5c) and employed a linear regression analysis. The obtained results show that there is a linear correlation between the theoretical concentrations and the calculated concentrations, with an R^2^ value of 0.9994 (Figure 5c). The ZA calibration curve (Figure 4) shows also that the linearity of the method is close to absolute, with an R^2^ value of 0.9967. A calibration curve is considered linear if R^2^ is over 0.98 [28].

#### 3.3.5. Signal-to-Noise Ratio

In order to obtain the signal-to-noise ratio (S/N), we used the MassHunter Qualitative Analysis software instructions, as specified in Section 2.3.3—Signal-to-Noise Ratio. The set time intervals were chosen before the ZA peak between 1.500 and 2.000 min and after the ZA peak between 3.200 and 3.700 min. This interval was saved in the method editor of MassHunter Qualitative Analysis software (Agilent Technologies, Inc., Santa Clara, CA, USA) and all the S/N values were calculated automatically. For all chromatograms, we obtained an S/N ratio of around 4.2, showing a higher recorded signal power than noise power.

#### 3.3.6. Limit of Detection and Limit of Quantification

LOD and LOQ for ZA quantitation, calculated according to the formulas in Section 2.3.3—Limit of Detection and Limit of Quantification, were 1.26 ng ZA/mL and 4.2 ng ZA/mL, respectively.

## 4. Conclusions

The complexed chemical species that develop between ZA and free calcium ions in simulated body fluid with high calcium were examined. By determining the insoluble complexed species formed between ZA and nearby calcium-free ions, we were able to develop an analytical method for identifying and quantifying low concentrations of ZA in calcium-rich media. The developed HPLC-ESI-MS method was validated using the following parameters: selectivity, precision, accuracy, linearity, signal-to-noise ratio, limit of detection and limit of quantification. The HPLC-ESI-MS method that we developed and validated is able to detect ZA amounts in the range of 0.1–0.5 ng/mL, and to precisely quantify it down to a concentration of 0.1 ng/mL. This method could be used to quantify low levels of ZA in the blood after long-term administration.

## Figures and Tables

**Table 1:** Data confirming the precision of the analytical method used (for 0.1 ng ZA/mL standard solution).

Sample	Rt	EIC Peak Area of *m*/*z* 273 [M + H]^+^ × 10^−4^	SD	RSD%	Start Rt	End Rt
ZA 0.1 ng/mL-amino-1	2.316	273.0715	0.0306	0.0112	2.123	3.185
ZA 0.1 ng/mL-amino-2	2.315	272.9948	0.0236	0.0086	2.09	3.169
ZA 0.1 ng/mL-amino-3	2.304	272.9493	0.0558	0.0204	2.094	3.189
ZA 0.1 ng/mL-amino-4	2.314	273.0887	0.0428	0.0157	2.121	3.184
ZA 0.1 ng/mL-amino-5	2.305	273.1494	0.0857	0.0314	2.064	3.175
ZA 0.1 ng/mL-amino-6	2.322	272.9155	0.0797	0.0292	2.097	3.176
ZA 0.1 ng/mL-amino-mean value		273.0282				

**Table 2:** Data recorded and calculated for accuracy determination.

Sample	Rt	EIC Peak Area of *m*/*z* 273 [M + H]^+^ × 10^−4^	SD	RSD%	Start Rt	End Rt
80%-ZA-amino-1	2.305	357.123	0.1118	0.0313	1.999	3.174
80%-ZA-amino-2	2.282	357.1992	0.0579	0.0162	2.073	3.184
80%-ZA-amino-3	2.288	357.4954	0.2758	0.0772	2.014	3.173
80%-ZA-amino-4	2.286	356.891	0.1393	0.0389	2.012	3.188
80%-ZA-amino-5	2.318	357.4781	0.1393	0.0389	2.061	3.188
80%-ZA-amino-6	2.317	357.4999	0.1547	0.0433	2.027	3.17
80%-ZA-amino-mean value		357.2811				
100%-ZA-amino-1	2.31	388.4991	0.1265	0.0326	2.068	3.179
100%-ZA-amino-2	2.379	388.4135	0.1871	0.0481	1.977	3.184
100%-ZA-amino-3	2.426	388.6965	0.0130	0.0034	2.008	3.183
100%-ZA-amino-4	2.428	389.0743	0.2802	0.0721	2.01	3.185
100%-ZA-amino-5	2.757	388.8763	0.1402	0.0361	2.097	3.175
100%-ZA-amino-6	2.934	388.5086	0.1198	0.0308	2.081	3.176
100%-ZA-amino-mean value		388.6781				
120%-ZA-amino-1	2.436	417.0019	0.0067	0.0016	2.05	3.177
120%-ZA-amino-2	2.436	417.8364	0.5834	0.1399	2.066	3.177
120%-ZA-amino-3	2.384	416.344	0.4719	0.1132	2.094	3.189
120%-ZA-amino-4	2.374	417.4411	0.3039	0.0729	2.084	3.179
120%-ZA-amino-5	2.369	417.1665	0.1097	0.0263	2.096	3.174
120%-ZA-amino-6	2.38	416.2783	0.5184	0.1243	2.058	3.185
120%-ZA-amino-mean value		417.0114				

## Data Availability

The data presented in this study are available on request from the corresponding author.
